# RETRACTION: Analgesic Efficacy of Local Infiltration Anaesthesia versus Femoral Nerve Block in Alleviating Postoperative Wound Pain Following Total Knee Arthroplasty: A Systematic Review and Meta‐Analysis

**DOI:** 10.1111/iwj.70536

**Published:** 2025-04-18

## Abstract

**RETRACTION:** YuD. , WuY. , HanS. , WangX. , and JiangL. , “Analgesic Efficacy of Local Infiltration Anaesthesia versus Femoral Nerve Block in Alleviating Postoperative Wound Pain Following Total Knee Arthroplasty: A Systematic Review and Meta‐Analysis,” International Wound Journal 21, no. 2 (2024): e14766, 10.1111/iwj.14766.38351465 PMC10864686

The above article, published online on 13 February 2024, in Wiley Online Library (http://onlinelibrary.wiley.com/), has been retracted by agreement between the journal Editor in Chief, Professor Keith Harding; and John Wiley & Sons Ltd. Following an investigation by the publisher, all parties have concluded that this article was accepted solely on the basis of a compromised peer review process. The editors have therefore decided to retract the article. The authors did not respond to our notice regarding the retraction.

**RETRACTION:** D. Yu , Y. Wu , S. Han , X. Wang , and L. Jiang , “Analgesic Efficacy of Local Infiltration Anaesthesia versus Femoral Nerve Block in Alleviating Postoperative Wound Pain Following Total Knee Arthroplasty: A Systematic Review and Meta‐Analysis,” International Wound Journal 21, no. 2 (2024): e14766, 10.1111/iwj.14766.38351465 PMC10864686

The above article, published online on 13 February 2024, in Wiley Online Library (http://onlinelibrary.wiley.com/), has been retracted by agreement between the journal Editor in Chief, Professor Keith Harding; and John Wiley & Sons Ltd. Following an investigation by the publisher, all parties have concluded that this article was accepted solely on the basis of a compromised peer review process. The editors have therefore decided to retract the article. The authors did not respond to our notice regarding the retraction.

